# Genome-Wide Identification, Expression Pattern Analysis and Evolution of the *Ces/Csl* Gene Superfamily in Pineapple (*Ananas comosus*)

**DOI:** 10.3390/plants8080275

**Published:** 2019-08-08

**Authors:** Shijiang Cao, Han Cheng, Jiashuo Zhang, Mohammad Aslam, Maokai Yan, Anqi Hu, Lili Lin, Simon Peter Ojolo, Heming Zhao, S.V.G.N. Priyadarshani, Yuan Yu, Guangqiu Cao, Yuan Qin

**Affiliations:** 1 College of Forestry, Fujian Agriculture and Forestry University, Fuzhou 350002, China; 2 Chinese Fir Engineering Technology Research Center under National Forestry and Grassland Administration, Fuzhou 350002, China; 3 Fujian Provincial Key Laboratory of Haixia Applied Plant Systems Biology, Fuzhou 350002, China; 4 Key Laboratory of Genetics, Breeding and Multiple Utilization of Crops, Ministry of Education, College of Crop Science, Fujian Agriculture and Forestry University, Fuzhou 350002, China; 5 College of Plant Protection, Fujian Agriculture and Forestry University, Fuzhou 350002, China; 6 College of Life Sciences, Fujian Agriculture and Forestry University, Fuzhou 350002, China; 7 FAFU-UCR Joint Center for Horticultural Biology and Metabolomics, Haixia Institute of Science and Technology, Fujian Agriculture and Forestry University, Fuzhou 350002, China; 8 State Key Laboratory for Conservation and Utilization of Subtropical Agro-Bioresources, Guangxi Key Lab of Sugarcane Biology, College of Agriculture, Guangxi University, Nanning 530004, China

**Keywords:** cellulose synthase gene, expression profile, RNA-Seq, *Ananas comosus*

## Abstract

The cellulose synthase (Ces) and cellulose synthase-like (Csl) gene families belonging to the cellulose synthase gene superfamily, are responsible for the biosynthesis of cellulose and hemicellulose of the plant cell wall, and play critical roles in plant development, growth and evolution. However, the *Ces/Csl* gene family remains to be characterized in pineapple, a highly valued and delicious tropical fruit. Here, we carried out genome-wide study and identified a total of seven *Ces* genes and 25 *Csl* genes in pineapple. Genomic features and phylogeny analysis of *Ces/Csl* genes were carried out, including phylogenetic tree, chromosomal locations, gene structures, and conserved motifs identification. In addition, we identified 32 pineapple *AcoCes/Csl* genes with 31 *Arabidopsis AtCes/Csl* genes as orthologs by the syntenic and phylogenetic approaches. Furthermore, a RNA-seq investigation exhibited the expression profile of several *AcoCes/Csl* genes in various tissues and multiple developmental stages. Collectively, we provided comprehensive information of the evolution and function of pineapple *Ces/Csl* gene superfamily, which would be useful for screening out and characterization of the putative genes responsible for tissue development in pineapple. The present study laid the foundation for future functional characterization of *Ces/Csl* genes in pineapple.

## 1. Introduction

The cell wall, as a key component of plant cell, plays vital roles in the whole process of plant growth. The plant cell synthesizes and deposits the wall polymers to adjust the architecture of the cell wall according to requirements and it also manages the physical properties of the cell wall. Generally, cell walls are made of the primary cell wall and the secondary cell wall. The primary cell wall is deposited during cell growth and the secondary cell wall is formed when an expansion ends. Polysaccharides including hemicellulose, cellulose and pectin and proteins are common components of cell walls [1]. Cellulose is the main component of primary and secondary plant cell walls. Cellulose displays huge tensile firmness to the cell wall. In nature, about 180 billion tons of cellulose is generated every year, which is an important renewable resource on the earth [2]. Cellulose synthesis captures a lot of interest and many reviews have highlighted the contributions toward the understanding of cellulose biosynthesis [2,3,4,5,6,7]. The study on the regulation mechanism of plant cell wall synthesis and modification has been one of the most important fields in plant development biology.

Cellulose synthase (CesA) genes encode cellulose which is consists of β-1, 4 linked glucan residue chains [8,9,10]. Cellulose synthesis is coordinated by the strict and complex transcriptional regulation system. Similar to CesA sequences, cellulose synthase-like (*Csl*) genes also make a gene family. The *Csl* genes are mainly responsible for the biosynthesis of hemicellulose [11,12] that with cellulose forms a matrix in the cell walls [13,14]. The *CesA* and *Csl* gene families constitute the cellulose synthase gene superfamily that is classified as glycoside transferase gene family (GT2) in the Carbohydrate-Active enZYmes Database (CAZy) database based on sequence similarity, GT2 consists of conserved and variable regions [15,16]. GT2 proteins possess a class-specific region (CSR) and a plant-conserved region as well as four conserved motifs (QxxRW, DD, DCD, and TED) to be responsible for binding to the substrate [17].

All CesA and Csl proteins have several transmembrane domains (TMs). At the N-terminal, two TMs are found and the remaining are found near the C-terminal which contains a hydrophilic intracellular region [12]. The maximum homology between CesA and Csl proteins occurs in the intracellular region. *Csl* and *CesA* genes have high sequence similarity, and their proteins have glycosyltransferase activity [15]. In addition, they contain an enzyme catalytic site for the motif D-D-D-QxxRW (D, Q, R, and W represent standard amino acid, while x depicts any amino acid) [18]. The main difference between Csl and CesA is that the Csl family lacks a zinc finger structure, which has a very conservative repetitive sequence CXXC (cysteine-X-X-cysteine). The sequence can bind to DNA and play an important role to maintain the stability of cellulose synthase complexes, and be involved in the interaction between the subunits [2]. Since Csl lacks a zinc finger structure, it has been suggested that hemicellulose synthesis probably does not require such a structure, and a single Csl protein may have the catalytic activity to form the main chain of hemicellulose.

The functions of CesAs and Csls have been well studied. In *Arabidopsis*, *AtCesA1*, *AtCesA3* and *AtCesA6,* genes function in the cellulose biosynthesis and composition of the primary cell wall [19]. In addition, loss-of-function mutants of *AtCesA3* and *AtCesA6* leaded to a decrease in cellulose [19,20,21]. The functional loss mutants of *AtCesA4*, *AtCesA7* and *AtCesA8* showed reduced cellulose content in the secondary cell wall, which was usually accompanied by changes in xylem structure. Further analysis revealed that *AtCesA4*, *AtCesA7* and *AtCesA8* co-expressed, and interacted together [22,23,24]. Csl consists of eight subfamilies (*CslA, CslB/HCslC, CslD, CslE, CslF, CslG and CslJ/M*), and the *CslD* subfamily is common in terrestrial plants. The *Csl* subfamilies show high similarity in sequence with cellulose synthase gene. So far, six, five and five *CslD* genes have been identified in *Arabidopsis*, rice and maize (*Zea mays*), respectively. Several *Csl* genes have been demonstrated to be directly responsible for the biosynthesis of cellulose in *Arabidopsis*, poplar (*Populus alba*) and rice (*Oryza sativa*). [25,26]. In rice, the *CslF* gene family plays a crucial role in cell wall formation and growth [26,27]. In *Tropaeolum majus*, it was found that one *TmCsl* was related to the synthesis of the 1, 4-glucan skeleton of xyloglucan (XyG), the main component of hemicellulose in the primary cell wall, and this protein is highly homologous to *Arabidopsis CslC4*. Mutants with the loss-of-function of *AtCslD1* and *AtCslD4* showed significantly reduced cellulose deposition in the cell wall of pollen tube, and the histological sequence from the cell walls of pollen tube was significantly broken, thus affecting the germination and growth of pollen tube and the transmissibility of male gametophyte [28]. All in all, CesA and Csl proteins play key roles in the plant growth and development.

Gram negative bacterium, *Acetobacter xylinus*, was first reported to contain a gene encoding cellulose synthase designated as BcsA [29,30,31]. The first plant CesA proteins were identified based on a homology search between bacterial BcsA and expressed sequence tags from cotton [2,29,30,31,32]. Recently, researchers on the *CesA* and *Csl* gene superfamily have been reported in various plant species, such as *Arabidopsis*, maize, wheat (*Triticum aestivum*), poplar, pine (*Pinus*), rice, eucalypt (*Eucalyptus tereticornis*) and alfalfa (*Medicago sativa*) [12,14,18,25,26,27,33,34,35,36,37,38,39,40,41,42]. However, the *CesA* and *Csl* have not been explored in pineapple, a highly valued tropical fruit with a gross production value reaching nine billion dollars annually and having outstanding nutritional and medicinal properties [43]. Recently, the genome of the pineapple was sequenced providing the opportunity to decipher the gene functions during pineapple developmental and stress response [44,45,46,47,48,49].

In this study, we identified *AcoCes/Csl* candidate genes using the reference genome of the pineapple, and analyzed the domain, motif and gene structure of *AcoCes/Csl* genes. We also studied the evolutionary relationship of *AcoCes/Csl* genes and analyzed the phylogenetic relationship of the *Ces/Csl* gene family between *Arabidopsis* (dicot) and rice (monocot). Furthermore, we studied the expression pattern of *Ces/Csl* genes in different pineapple tissues and developmental stages. Our results provide the key information for further evaluation and functional characterization of *AcoCes/Csl* genes in pineapple.

## 2. Results

### 2.1. Identification of Ces /Csl Genes in Pineapple

**Figure 1:**
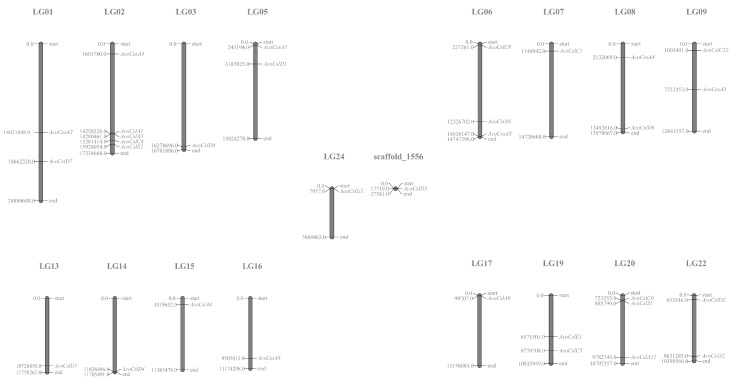
Distribution of *AcoCes/Csl* genes on the pineapple genome. The gene start points are shown on the chromosome. Thirty-two *Ces/Csl* genes of pineapple were mapped to different chromosomes using MapChart. Only those chromosomes bearing *AcoCes/Csl* are represented. The prefix ‘Aco’ indicates *Ananas comosus*.

We identified a total of 32 candidate genes from the pineapple genome. The 32 proteins were grouped into seven subfamilies, including AcoCesA, AcoCslA, AcoCslC, AcoCslD, AcoCslE, AcoCslG and AcoCslJ according to the phylogenetic relationships with Arabidopsis and rice (Table 1). CslD had the maximum number of members (with eight genes) among the identified subfamilies. The smallest subfamilies were CslG and CslJ, both containing only one member. The gene distribution in chromosomes was showed in Figure 1. These genes were mapped on 17 pineapple chromosomes and one scaffold. Chr3 possessed five genes, Chr20 contained three genes, seven chromosomes each contained two genes, and eight chromosomes each contained one gene. Moreover, the remaining one gene was located on scaffold 1556.

Characteristics of the 32 *Ces/Csl* genes were shown in Table 1. Genomic DNA size of genes in this gene superfamily varied from 2630 kb (*AcoCslC4*) to 20,041 kb (*AcoCslA4*). The average length of these genes was 8270 kb. Genomic DNA length did not change much in the *CesA* subfamily rather than *CesA3*, *CslC*, *CslD* and *ClsE* subfamilies. The numbers of predicted amino acids ranged from 536 aa (*AcoCslA2*) to 1615 aa (*AcoCslG1*) with the corresponding molecular weight varied from 60.9 kDa to 178.6 kDa. The *CslC* subfamily showed great divergence in terms of amino acid length (536 aa–1184 aa), which differed from the other subfamilies. The *CesA* and *CslD* subfamilies constituted similar amino acid length. The predicted isoelectric points varied from 5.93 (*AcoCesA8*) to 9.19 (*AcoCslA2*). Besides these, the minimum intron number was two that was found in *CslD* subfamily including *AcoCslD2*, *AcoCslD3*, *AcoCslD5* *and* *AcoCslD8*. The CslA subfamily has the maximum intron number including *AcoCslA1* (19) and *AcoCslA4* (20). The intron number of *CesA*, *CslC*, *CslD* and *CslE* subfamily changed little, especially for *CslE* that two members had the same intron number. The intron number of *CslA* subfamily varied from eight to 20.

### 2.2. Phylogenetic Analysis of the Pineapple Ces/Csl Gene Superfamily

Based on the phylogenetic distribution, pineapple Ces/Csl proteins could be divided into five subgroups (Figure S1), including I, II, III, IV and V. Subgroup I possessed all the CslA proteins, Subgroup II contained all the CslC proteins, Subgroup III consisted the CslE, CslG and CslJ proteins, Subgroup IV contained all the CesA proteins and subgroup V consisted all the CslD proteins.

**Figure 2:**
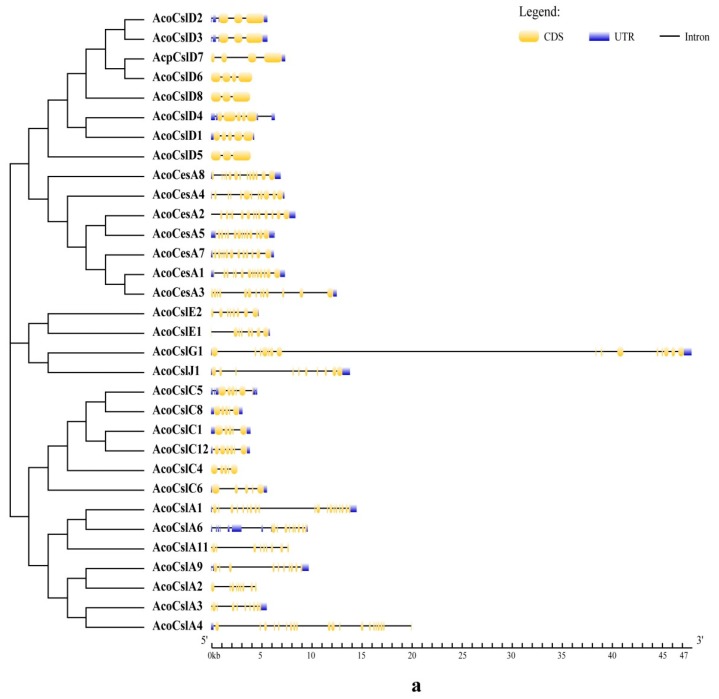

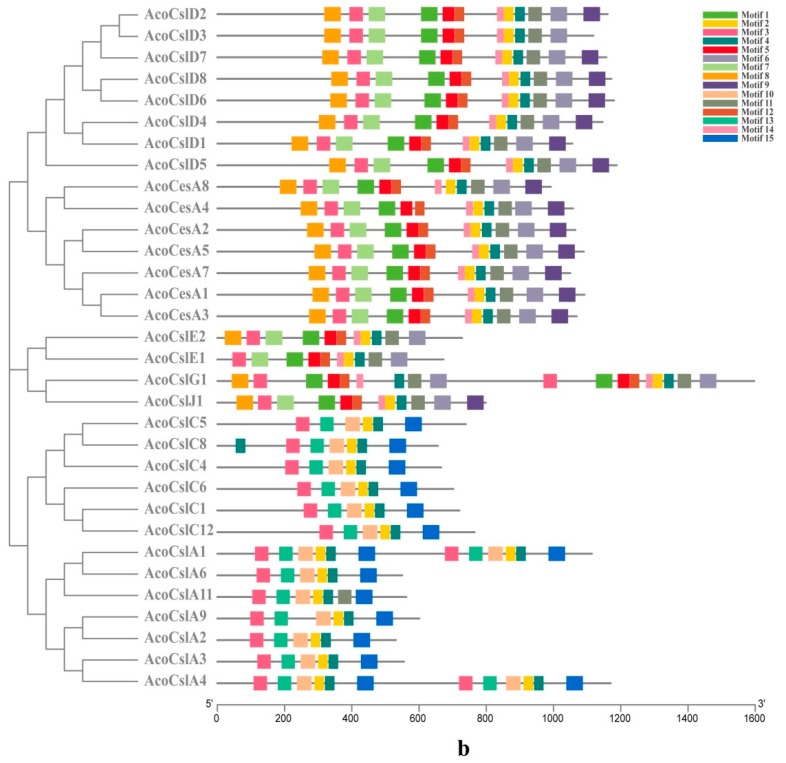
(**a**) Exon-intron structure of pineapple *Ces/Csl* genes. Bule boxes indicated untranslated 5’- and 3-regions; yellow boxes indicated exons; black lines indicate introns. The prefix ‘Aco’ indicates *Ananas comosus*. (**b**) Motif analysis of the pineapple Ces/Csl protein. Motifs with specific colors can be found on the respective *AcoCes/Csl* protein. The combined phylogenetic trees of *AcoCes/Csl* subfamily on the left panel. The motifs of corresponding proteins are shown on the right panel with specific colors on behalf of different motifs using the Multiple Em for Motif Elicitation (MEME). The order of the motifs corresponds to their position within individual protein sequences. Prefix ‘Aco’ indicates *Ananas comosus*.

The related sister pairs appeared in the joint phylogenetic tree (such as *AcoCslA3* and *AcoCslA4*) and triplets (such as *AcoCslA1*, *AcoCslA6* and *AcoCslA11*) [50,51]. We found, seven sister pairs and five triplets among the *AcoCes/Csl* gene families. The similar intron-exon structure existed in the sister pairs or triplets (Figure 2a) validating the phylogenetic results. The structural diversities among the *AcoCes/Csl* genes suggested that the gene family could be showing functional divergence.

**Figure 3:**
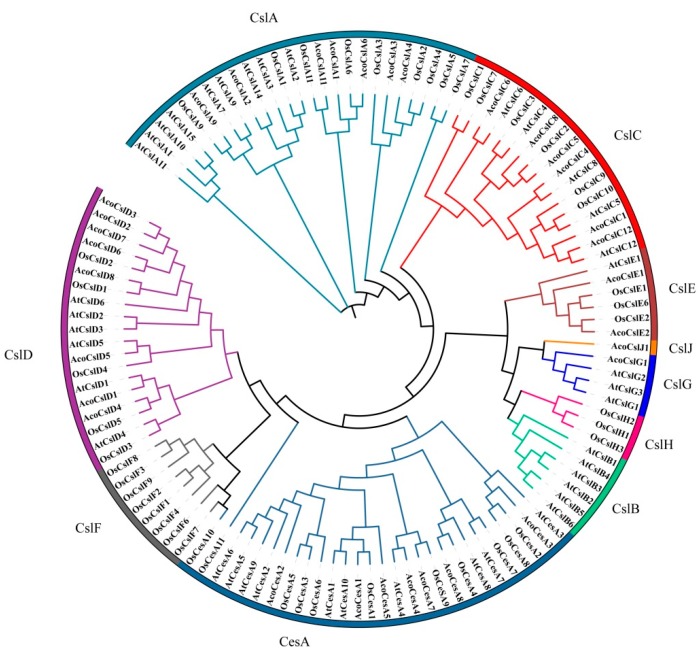
Phylogenetic tree depicting the relationships among Ces/Csl proteins from pineapple, *Arabidopsis* and rice. All the Ces/Csl protein sequences were aligned and phylogenetic tree was constructed using MEGA 7.0. The different colored arcs indicated different subgroups. Prefix ‘Ath’, Osa, and ‘Aco’ indicate Ces/Csl proteins from *Arabidopsis, Oryza sativa,* and *Ananas comosus*, respectively.

Based on the full-length protein sequences, we constructed a multi-species phylogenetic tree of *Ces/Csls* from *Arabidopsis*, pineapple and rice, in order to investigate the functional associations and evolutionary relationships of pineapple *Ces/Csl* genes (Figure 3). The gene names and IDs of *Ces/Csl* from *Arabidopsis* and rice were presented in Table S1. The phylogenetic analysis suggested that *Ces/Csl* can be grouped into 10 subfamilies: *CslD*, *CslF*, *CesA*, *CslB*, *CslH*, *CslG*, *CslJ*, *CslE*, *CslC* and *CslA*. The *CesA* subfamily was the greatest subfamily, with seven pineapple *CesA* genes, 10 *Arabidopsis* genes, nine rice genes, accounting for 21% of the total *Ces/Csl* genes. *CslA* was the second largest subfamily, having seven genes from pineapple, nine from *Arabidopsis*, and nine from rice genes. The smallest subfamily was *CslJ* with only one pineapple gene. No pineapple gene was found in *CslB*, *CslF* and *CslH*. *Arabidopsis* had six *CslB* genes, three *CslH* and eight *CslF* genes were found in rice.

According to the phylogenetic tree, we identified 10 sister gene pairs between pineapple and rice; *AcoCesA7/OsCesA9*, *AcoCesA8/OsCesA4*, *AcoCesA1/OsCesA1*, *AcoCslC5/OsCslC2*, *AcoCslA6/OsCslA6*, *AcoCslA11/OsCslA11*, *AcoCslA9/OsCslA9*, *AcoCslD6/OsCslD2*, *AcoCslD8/OsCslD1*, *AcoCslD4/OsCslD5*, six sister gene pairs between pineapple and Arabidopsis; *AcoCesA4/AtCesA8*, *AcoCslC12/AtCslC12*, *AcoCslC4/AtCslC8*, *AcoCslC6/AtCslC6*, *AcoCslD5/AtCslD5*, *AcoCslD1/AtCslD1* Four triplets were found between pineapple and rice; *AcoCesA3/OsCesA2/OsCesA8*, *AcoCslA4/OsCslA2/OsCslA4*, *AcoCslC8/AcoCslC5/OsCslC2* and *AcoCslA6/OsCslA6*/*AcoCslA1*, and one triplet was identified between pineapple and Arabidopsis; *AcoCslC1/AcoCslC12/AtCslC12*.

### 2.3. Gene Structure Analysis and Conserved Motif Identification

The evolutionary aspect and structural diversity of the *Ces/Csl* genes in pineapple were explored by studying the exon-intron organization. The difference in the gene architecture such as number of exons, introns and the lengths of untranslated region (UTR) among gene pairs suggest that the paralogs could be having separate roles during pineapple growth and development [52]. The number of exons in *AcoCes/Csl* genes varied from three to 20. *AcoCslA4* had the maximum exons, whereas *AcoCslD8* and *AcoCslD5* had only three exons and three genes (*AcoCslD6*, *AcoCslD8* and *AcoCslD5*) had no UTR. To further reveal the diversification of *AcoCes/Csl* gene family in pineapple, we predicted the putative motifs by MEME with the default setting [53]. In total, 15 motifs were identified in the *AcoCes/Csl* gene family. *AcoCslD* and *AcoCesA* subgroup had 12 same motifs, while *AcoCslD* lacked in the motif 9. Most of the *AcoCslA* and *AcoCslC* subgroup contained six same motifs, except for *AcoCslC8*, *AcoCslA1*, *AcoCslA11* and *AcoCslA4* (Figure 2b).

### 2.4. Synteny Analysis of Pineapple Ces/Csl Genes

**Figure 4:**
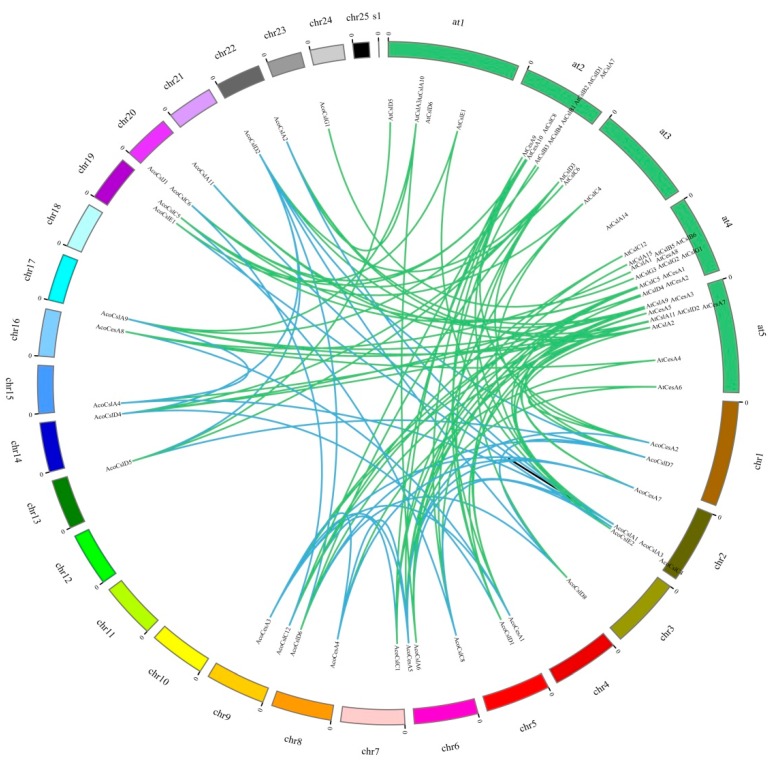
Synteny analysis between pineapple and *Arabidopsis Ces/Csl* genes. Chromosomes and scaffolds of pineapple and *Arabidopsis* are shown in different colors and in partial circles. Colored curves indicate the syntenic relationships between pineapple and *Arabidopsis Ces/Csl* genes. The prefix ‘Aco’ indicates *Ananas comosus*.

Segmental and tandem duplication gene pairs (identity ≥ 50%) of the *Ces/Csl* gene family were studied to test the duplication effect. One tandem duplication pair (*AcoCesA3* and *AcoCesA7*), which showed a high coding sequence similarity, was distributed closely on the chromosome 2. In addition, we identified 34 pairs of segmental duplication events where each pair of gene is situated at separate chromosomes in pineapple *Ces/Csl* genes, such as *AcoCesA8/AcoCesA1*, *AcoCslD1/AcoCslD4*, *AcoCslC5/AcoCslC8* (Figure 4). Overall, our results showed that tandem and segmental duplication resulted in the expansion of the pineapple *Ces/Csl* gene family.

Additionally, the syntenic relationship between pineapple and *Arabidopsis* was also investigated to study the evolution of pineapple *Ces/Csl* genes. Two types of pineapple *Ces/Csl* genes were found in synteny analysis. The first type of pineapple *Ces/Csl* genes was that a pineapple *Ces/Csl* gene related with a single *Arabidopsis* gene viz., *AcoCslA4-AtCslA9* and *AcoCesA4-AtCesA4*. The second type is that a pineapple *Ces/Csl* gene associated with multiple *Arabidopsis* genes, for example, *AcoCslC8-AtCslC8*, *AtCslC5*, *AtCslC4*; *AcoCesA1-AtCesA1*, *AtCesA3*, *AtCesA10*. The more elaborated information is given in Supplemental Table S2.

### 2.5. Expression Patterns of AcoCes/Csl Genes in Four Different Tissues

The transcriptome analysis was carried out to understand tissue-specific expression patterns of *AcoCes/Csl* genes. From the RNA-seq data the expressions of 32 *AcoCes/Csl* genes in flower, fruit, leaf and root were studied using their fragments per kilobase of exon model per million mapped reads (FPKM) values [44].

**Figure 5:**
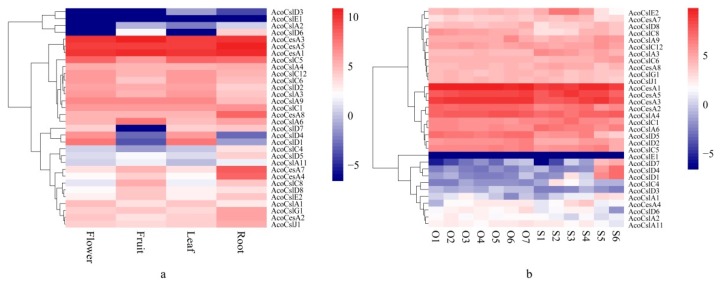
Organ-specific expression profiles of the pineapple *AcoCes/Csl* genes. (**a**) Expression profiles of *AcoCes/Csl* genes in flower, fruit, root and leaf of pineapple. (**b**) Expression patterns of *AcoCes/Csl* genes in developing ovule and stamen of pineapple. Hierarchical clustering of expression profiles of pineapple *Ces/Csl* genes in different organs and developmental stages. Red colors indicate high levels of transcript abundance, and blue colors indicate low transcript abundance. The color scale is shown at right side of the figure. Sample details are mentioned at the bottom of each lane: Ovule O1–O7, stamen S1–S6. The number after different tissues name refers to the different stage. Prefix ‘Aco’ indicates Ananas comosus.

**Figure 6:**
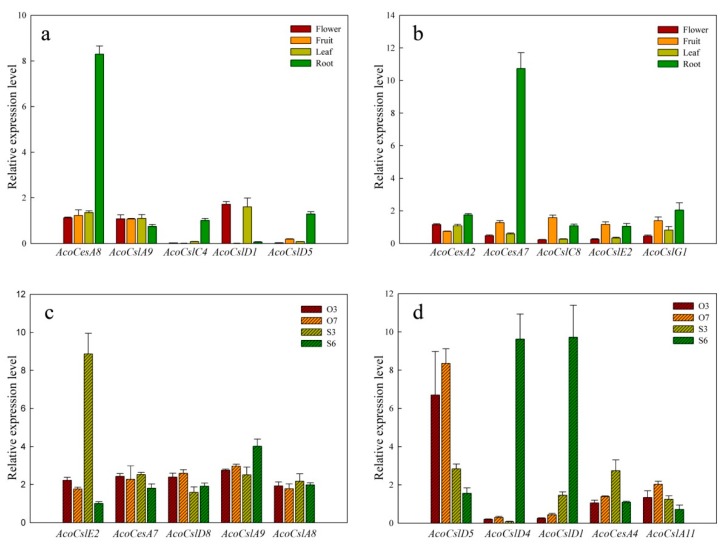
Expression profiles of the pineapple *Ces/Csl* genes in different tissues. (**a**,**b**) qPCR expression profiles of *AcoCes/Csl* genes in flower, fruit, root and leaf of pineapple. (**c**,**d**) qPCR expression pattern of *AcoCes/Csl* genes in developing ovule and stamen of pineapple. Sample details are mentioned at the top right corner: Ovule O3 and O7, stamen S3 and S6. The number after the different samples name refers to the different developmental stages. The gene details are mentioned at the bottom of each lane. Data were normalized to the EF1a gene. Vertical bars indicate standard error (SE). All experiments were performed with three technical and three biological repeats. The prefix ‘Aco’ indicates *Ananas comosus*.

As showed in Figure 5a, *AcoCslD3*, *AcoCslE1*, *AcoCslA2* and *AcoCslD2* were expressed in all sampled tissues at very low levels, implying that those genes might be expressed under special conditions or in other non-sampled pineapple tissues. Transcript levels of *AcoCesA3*, *AcoCesA5*, *AcoCesA1* and *AcoCslC5* were similar, showing very high expression in all the tested tissues, which suggested that these genes might be playing a crucial role in the plant development. Similar expression profiles were found in *AcoCslA4*, *AcoCslC12*, *AcoCslC6*, *AcoCslD2*, *AcoCslA3* and *AcoCslA3* with moderate and even expression level in four tissues. The expression level of *AcoCslD4* and *AcoCslD1* were higher in flower and leaf than fruit and root, indicating that the two genes may be responding in the growth of flower and leaf. Furthermore, *AcoCesA4*, *AcoCesA7* and *AcoCesA8*, showed high root-specific expression, suggesting these genes may be working during the root development. The remaining genes showed similar expression pattern. The RNA-seq data was further verified using qRT-PCR. For qRT-PCR, 10 genes (*AcoCesA8*, *AcoCslA9*, *AcoCslC4*, *AcoCslD1*, *AcoCslD5*, *AcoCesA2*, *AcoCesA7*, *AcoCslC8*, *AcoCslE2* and *AcoCslG1*) were selected. AcoCesA8 and AcoCesA7 showed higher expression in root and relative lower expression than the other three tissues. *AcoCesC4* and *AcoCesD5* also exhibited root-specific expression, but barely expressed it in flower, fruit and leaf (Figure 6a,b). However, for *AcoCesD5*, the higher expression was found in flower and leaf, but did not express it in the root and fruit. The other five candidate genes expressed showed no significant difference between four tissues which were consistent with the results from RNA seq data.

### 2.6. Expression of AcoCes/Csl Genes during Gametophyte Development

The roles of *AcoCes/Csl* genes in pineapple were further studied to understand their roles in reproductive development. The expression patterns of 32 *AcoCes/Csl* in ovules and stamens were investigated using transcriptome data. As showed in Figure 5b, the expression profiles revealed that 10 *AcoCes/Csl* genes (*AcoCesA1*, *AcoCesA5*, *AcoCesA3*, *AcoCesA2*, *AcoCslA4*, *AcoCslC1*, *AcoCslA5*, *AcoCslD2*, *AcoCslD2*, *AcoCslD5* and *AcoCslC5*) were expressed highly in various stages of ovule and six stages of stamens, implying they may be performing crucial role in the formation reproductive organs. Eleven *AcoCes/Csl* genes were expressed moderately and evenly in all tested tissues. *AcoCslD7*, *AcoCslD4* and *AcoCslD1* showed similar expression level that had higher expression in the stage 5 and stage 6 of stamens than other tissues. Seven *AcoCes/Csl* genes showed low expression levels in every tissue. *AcoCslE1* had the lowest expression in all the tissues. The different stages in pineapple reproductive organs were selected as reported earlier [54]. To further validate these results, 10 genes were selected (*AcoCslE2*, *AcoCesA7*, *AcoCslD8*, *AcoCslA9*, *AcoCslA8*, *AcoCslD5*, *AcoCesD4*, *AcoCesD1*, *AcoCesA4* and *AcoCslA12*) to perform qRT-PCR analysis. *AcoCslE2* expressed higher in stage 3 stamens than other tissues. *AcoCslD5* showed higher expression in stamens than ovules. *AcoCesD4* and *AcoCesD1* exhibited highest expression in stage 6 of stamens, but barely expressed in ovules and stage 3 of stamens (Figure 6c,d). The other 6 candidate genes expressed showed no significant difference between four tissues which were also coincided the results from RNA seq data.

## 3. Discussion

Based on the *Arabidopsis* database, the cellulose synthase superfamily was initially divided into the *CesA* family and six *Csl* families including *A*, *B*, *C*, *D*, *E* and *G* [12]. They belong to the integral membrane proteins, CesA proteins are located in the plasma membrane, however CslB, CslG and CslE are believed to locate in the Golgi [2]. The conservation of intron-exon structure exists in *CesA*, *CslB*, *CslG* and *CslE*, but not in other three families [2]. The CesA is responsible for the synthesis of cellulose, and the Csl participates in the synthesis of hemicellulose [12]. Three specific lineages including *CslF* [26], *CslH* [26] and *CslJ* [55] have been identified in the Poaceae. All of them have functions in the biosynthesis of the cell wall, and the three lineages have a wide distribution in the Poaceae but a narrow distribution in other angiosperms [55,56,57]. Base on the available genome sequence *CslF*, which was phylogenetically originated from the oldest family, *CslD* is presented in the graminid and restiid families [13]. However, no *CslF* genes were found in pineapple. *CslH*, which showed the monocot-specific sister branch to *CslB*, was not found in our study. While the *CslH* genes are involved in the synthesis of (1,3; 1,4)-β-glucan [57], the function of the CslB genes were not found. The *CslJ* was reported in barley, mediating the synthesis of the cell wall polysaccharide [13,55]. Even the *CslJ* genes were widely found in monocots, but only one was identified in our study. The *CslM* was discovered to form a reciprocally monophyletic eudicot-monocot grouping with the *CslJ* clade. However, heterologous expression of the grape *VvCslM* (*Vitis vinifera*) is unable to produce any detectable signs, as shown in Table 1, 4-β-glucan [13]. The *CslM* and *CslJ* branches families were different in evolutionary histories, therefore *CslJ* lineage should be monocot-specific and *CslM* lineage is eudicot-specific [13].

Based on the *Arabidopsis* database, the cellulose synthase superfamily was initially divided into the *CesA* family and six *Csl* families including *A*, *B*, *C*, *D*, *E* and *G* [12]. They belong to the integral membrane proteins, CesA proteins are located in the plasma membrane, however CslB, CslG and CslE are believed to locate in the Golgi [2]. The conservation of intron-exon structure exists in *CesA*, *CslB*, *CslG* and *CslE*, but not in other three families [2]. The CesA is responsible for the synthesis of cellulose, and the Csl participates in the synthesis of hemicellulose [12]. Three specific lineages including *CslF* [26], *CslH* [26] and *CslJ* [55] have been identified in the Poaceae. All of them have functions in the biosynthesis of the cell wall, and the three lineages have a wide distribution in the Poaceae but a narrow distribution in other angiosperms [55,56,57]. Base on the available genome sequence *CslF*, which was phylogenetically originated from the oldest family, *CslD* is presented in the graminid and restiid families [13]. However, no *CslF* genes were found in pineapple. *CslH*, which showed the monocot-specific sister branch to *CslB*, was not found in our study. While the *CslH* genes are involved in the synthesis of (1,3; 1,4)-β-glucan [57], the function of the CslB genes were not found. The *CslJ* was reported in barley, mediating the synthesis of the cell wall polysaccharide [13,55]. Even the *CslJ* genes were widely found in monocots, but only one was identified in our study. The *CslM* was discovered to form a reciprocally monophyletic eudicot-monocot grouping with the *CslJ* clade. However, heterologous expression of the grape *VvCslM* (*Vitis vinifera*) is unable to produce any detectable signs, as shown in Table 1, 4-β-glucan [13]. The *CslM* and *CslJ* branches families were different in evolutionary histories, therefore *CslJ* lineage should be monocot-specific and *CslM* lineage is eudicot-specific [13].

Early publications revealed that the *CslD* genes mediated functions in tip growth [25,58,59]. In this study, *AcoCslD1* and *AcoCslD4* had very high expression levels in the developing stamen of pineapple, suggesting that *AcoCslDs* may regulate stamen development. The plant Ces/Csl superfamily perhaps comes from cyanobacteria by endosymbiotic transferring. The putative *CesA* genes in cyanobacteria *Anabaena* spp. exhibited homology to that shown from the previously reported plants [60]. The *CesA* lineage in a marine cyanobacterium (*Synechoccus* spp.) existed monophyletic to the embryophyte CesA clades. At present, the phylogenetic analysis divided the superfamily into two distinct evolutionary branches, the *CslA* and *CslC* clades and the *CesA* and *CslB/D/E/F/G/H/M* lineages [13]. The *CslA/C* genes represented an independent lineage to *CesA* and *CslB/D/E/F/G/H/J* lineage, probably being originated from a different endosymbiotic transfer. The *CslA* was more similar to *CslC* than bacterial *CesA*, and the *CslA/C* proteins were smaller than bacterial *CesA* protein [60,61]. Unlike CslCs, some CslAs showed mannan synthase activity [62]. In pineapple, the *CslA/CslC* lineages had seven and six members, respectively. It is suggested that *CslA* genes mediate the biosynthesis of mannan [63] and *CslC* genes are responsible for the biosynthesis of xyloglucan [62,64,65]. However, not all the genes from the CslA and CslC clades were in participation with the biosynthesis of mannan or xyloglucan. Two *AcoCslE* and one *AcoCslG* were identified in the pineapple, but no clear functions with respect to the types of synthesized polysaccharides have been assigned to these genes. The gene architecture differences lead to functional diversification or functional redundancy. It is possible that these functional redundant genes are marching to pseudogenes due to lack of selective stress. We did observe that one gene (*AcoCslE1*) had extremely low expression in all of the tested samples, indicating that this gene is likely a peseudogene without evidence of function after divergence with other family members. In addition, we found that 26 members of the superfamily aggregate in sub-telomeric regions of the chromosomes, however, the reason is not clear so far.

The pineapple is one of the nutrient-rich tropical fruits, containing lots of nutrients including vitamin C, vitamin B6 and folate, as well as dietary fiber. The fiber is divided into soluble type and insoluble type. The soluble fiber comes from the inside of plant cells, reducing the blood sugar and decreasing cholesterol levels by binding to the cholesterol. The insoluble fiber originates from the cell walls of plant cells and can bind to water, making the stool softer, speeding up its movement through the digestive tract and decreases the risks for hemorrhoids, diverticulosis and constipation. The content and quality of fiber is regulated by *Ces/Csl* genes. The research on this gene family is very useful for biotechnology to improve the quality and yield of pineapple.

In summary, our identifications of the *AcoCes/Csl* gene families provide useful information to understand the biosynthetic mechanisms of (1,3; 1,4)-β-glucan in pineapple and lay the foundation for studying the origin of cell wall polysaccharides. Furthermore, the *AcoCes/Csl* genes pave the way to further functional identification and can be candidate genes for quality improvement of pineapple in future works.

## 4. Materials and Methods

### 4.1. Identification of Ces/Csl in Pineapple

The Ces/Csl amino acid sequences of *Arabidopsis* and *Oryza sativa* were downloaded from The Arabidopsis Information Resource (TAIR) (http://www.Arabidopsis.org) and the Rice Genome Annotation Project (http://rice.plantbiology.msu.edu/index.shtml). To identify the pineapple *Ces/Csl* genes, we used the *Arabidopsis* Ces/Csl amino acid sequences to search pineapple proteome with Basic Local Alignment Search Tool (BLAST-P) and we downloaded the hidden Markov model (HMM) profiles of the cellulose synthase (PF03552) domain from the pfam database (http://pfam.xfam.org/). Then we used the HMM profiles to search the pineapple proteome database through the hmm search program with the e-value set 0.01. We used Simple Modular Architecture Research Tool (SMART) [66] to verify these sequences we obtained last step, and deleted the redundant sequences. The rest of the Ces/Csl sequences were subjected to the phylogenetic analysis. We used Multiple Sequence Comparison by Log-Expectation (MUSCLE) 3.7 [67] with default setting and performed multiple alignments with Ces/Csls sequences from pineapple, *Arabidopsis* and rice.

### 4.2. Physicochemical Properties and Phylogenetic Analysis

To further understand the physicochemical properties, we used ExPASy (http://web.expasy.org/compute_pi/) to predict the isoelectric point (PI) and molecular weight (MW) of pineapple Ces/Csl amino acid sequences. We constructed the phylogenetic tree by MEGA 7 [68] through the maximum likelihood (ML) method with a bootstrap option of n = 1000 and the pairwise deletion of gaps.

### 4.3. Conserved Motifs Analysis of Pineapple Ces/Csl Protein

The MEME program (http://meme-suite.org/) was used to find the conserved motifs of pineapple Ces/Csl proteins with the motifs number set 15, and other options were default.

### 4.4. Chromosome Localization and Gene Structural Analysis of Pineapple Ces/Csl Genes

We downloaded the information of chromosome localization of *AcoCes/Csl* genes from Phytozome. The information was visualized by MapChart, including the localization and the length in corresponding chromosomes. In addition, the online gene structure display server (http://gsds.cbi.pku.edu.cn/) [69] was used to visualize the *Ces/Csl* genes structure information about the quantity and distribution of exon and intron.

### 4.5. Synteny Analysis of Pineapple Ces/Csl Genes

We first used blastp program to search homolog pairs between pineapple and Arabidopsis. After that, MCSCANX was used to identify synteny block with default parameter, which means that at least 5 genes should be preserved in a collinear block [70].

### 4.6. RNA-Seq and qRT-PCR

The different pineapple tissues including flower, fruits, leaf and root, and ovule and stamen from different development stages (MD2) were selected according to the previous method [71]. The total RNA was isolated using RNA extraction kit (Omega Bio-Tek, Shanghai, China). The cDNA libraries were established using the NEBNext Ultra™ RNA Library Prep Kit for Illumina (NEB) according to the manufacturer’s protocol. The qualified libraries were sequenced on the Hiseq2500 machine (NEBNext RNA-Seq data (SRA315090) of different tissues were downloaded from the National Center for Biotechnology Information (NCBI) database [44]. The trimmed pair-end reads of all tissues were aligned to pineapple genome by using TopHat v2.1.1 with default parameter settings. The FPKM values were estimated and further processed by Cufflinks v2.2.1 software. qRT-PCR was employed using the SYBR Taq II (TakaRa, China) and the program was as follows: 94 °C for 25 s; 39 cycles of 94 °C for 5 s and 60 °C for 40 s; 94 °C for 20 s. All the experiments were carried out with three technical and three biological replicates.

## 5. Conclusions

The *Ces/Csl* gene superfamily plays a critical role in the biosynthesis of cellulose and hemicellulos, however, information about the pineapple *Ces/Csl* gene family remains elusive in pineapple. Here, we identified 32 *AcoCes/Csl* genes in the pineapple which could be divided into five groups. We also studied the basic features including isoelectric point, molecular weight, transmembrane domains, gene structure, chromosome location, phylogenetic analysis and the syntenic relationship of the 32 pineapple *Ces/Csl* genes compared to *Arabidopsis*. Gene expression profiles showed they could be playing necessary roles in the development of reproductive organs. Overall, the studies of pineapple *AcoCes/Csl* genes present important information for functional study and future pineapple research.

## Figures and Tables

**Table 1:** Properties of the *Ces/Csl* gene superfamily in pineapple.

Gene Name	Gene ID	Chr	Length	Amino Acids	Introns	Exons	Isoelectric Point (pI)	Molecular Weight (MW)
*AcoCesA1*	Aco014283	LG05	7376	1104	13	14	6.72	122.4
*AcoCesA2*	Aco018229	LG01	8424	1077	13	14	7.92	120.7
*AcoCesA3*	Aco024230	LG09	12,555	1081	12	13	8.31	119.6
*AcoCesA4*	Aco014585	LG08	7332	1070	11	12	7.61	119.9
*AcoCesA5*	Aco018552	LG06	6358	1102	13	14	7.04	123.2
*AcoCesA7*	Aco012076	LG02	6278	1062	12	13	6.33	118.9
*AcoCesA8*	Aco006039	LG16	6951	1003	12	13	5.93	111.7
*AcoCslA1*	Aco001096	LG02	14,534	1126	19	19	8.99	127.2
*AcoCslA2*	Aco006900	LG22	4546	536	8	9	9.19	60.9
*AcoCslA3*	Aco001095	LG02	5559	560	9	10	8.8	62.9
*AcoCslA4*	Aco004149	LG15	20,041	1184	20	20	8.8	133.1
*AcoCslA6*	Aco002889	LG06	9648	555	15	9	8.56	63
*AcoCslA9*	Aco016682	LG17	9750	607	10	10	9.16	68.4.
*AcoCslA11*	Aco014689	LG20	7773	568	9	10	7.83	64.1
*AcoCslC1*	Aco004974	LG07	3939	727	4	5	8.4	81.1
*AcoCslC4*	Aco000968	LG02	2630	673	4	5	8.85	77.1
*AcoCslC5*	Aco008242	LG19	4607	747	8	6	8.86	84.7
*AcoCslC6*	Aco013494	LG20	5582	709	4	5	8.67	78.7
*AcoCslC8*	Aco011603	LG06	3136	663	4	5	8.99	74.8
*AcoCslC12*	Aco008598	LG09	3881	773	6	7	6.15	85.7
*AcoCslD1*	Aco004435	LG05	4309	1068	4	5	7.62	116.9
*AcoCslD2*	Aco015969	LG22	5627	1174	4	3	7.49	129.2
*AcoCslD3*	Aco030607	scaffold_1556	5631	1131	4	3	6.74	124.7
*AcoCslD4*	Aco017129	LG14	6362	1159	6	5	6.46	127.5
*AcoCslD5*	Aco013738	LG13	3953	1201	2	3	8.45	130.9
*AcoCslD6*	Aco016995	LG08	4094	1193	4	5	7.9	130.5
*AcoCslD7*	Aco025070	LG01	7403	1170	3	4	6.7	128.9
*AcoCslD8*	Aco017291	LG03	3892	1185	2	3	7.08	130.2
*AcoCslE1*	Aco026335	LG19	5869	680	7	8	6.99	76.2
*AcoCslE2*	Aco000884	LG02	4750	736	7	8	8.02	81.9
*AcoCslG1*	Aco013153	LG24	47,982	1615	16	17	7.92	178.6
*AcoCslJ1*	Aco013513	LG20	13,869	807	9	10	8.91	89.2

## Supplementary Materials

Supplementary materials can be found at https://www.mdpi.com/2223-7747/8/8/275/s1. Figure S1: Phylogenetic tree depicting the relation between pineapple *Ces/Csl* genes, Table S1: The genes of Ces/Csl in *Arabidopsis* and rice, Table S2: Synteny block of Ces/Csl genes between pineapple and *Arabidopsis* genomes.

## Abbreviations

Aco	*Ananas comosus*
At	*Arabidopsis thaliana*
Ces	Cellulose synthase
Csl	Cellulose synthase-like
