# Supplementary material for: Genome-Wide Identification, Expression Pattern Analysis and Evolution of the Ces/Csl Gene Superfamily in Pineapple (Ananas comosus)
Source: Plants (Basel). 2019 Aug 8;8(8):275. doi: 10.3390/plants8080275 (PMC6724413; doi:10.3390/plants8080275)
Supplement: Supplementary file 1 [file plants-08-00275-s001.zip › plants-546538-supplementary-final/Figure S1.docx]

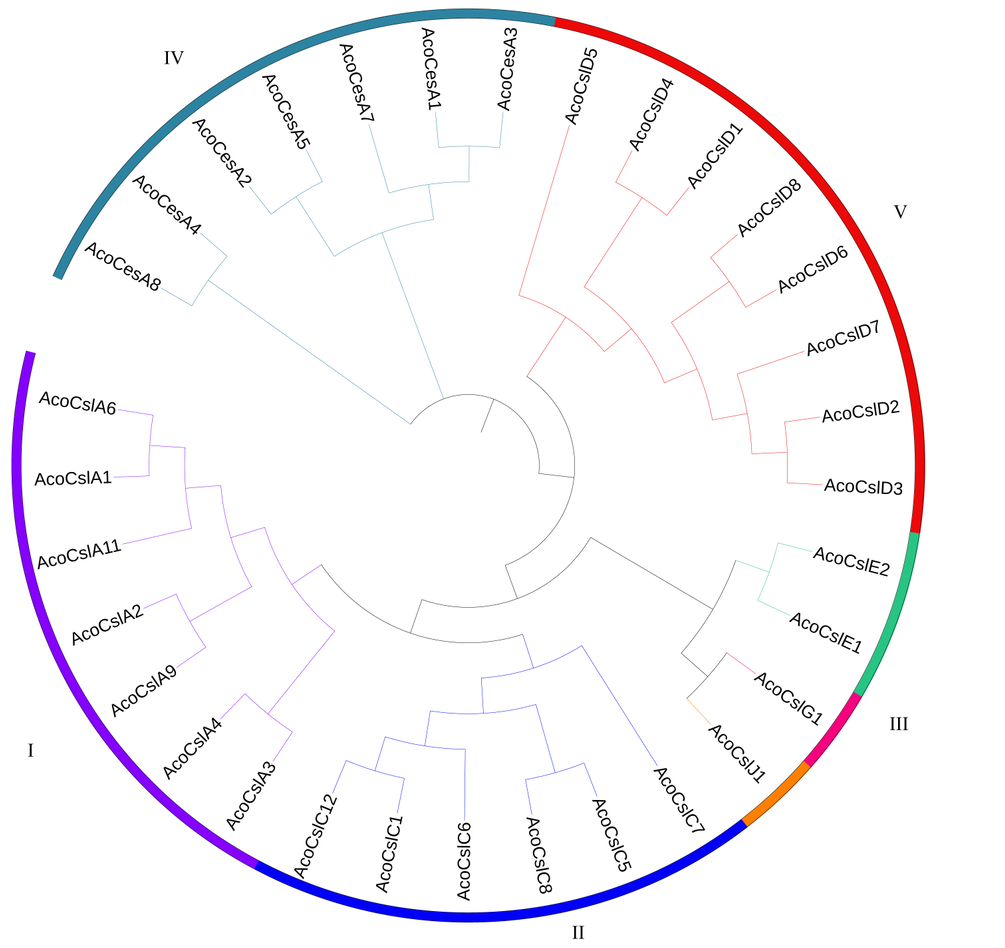


Figure S1: Phylogenetic tree depicting the relation between pineapple *Ces/Csl* genes. Different color arc represent different subgroups.
